# Use of Cigarettes and E-Cigarettes and Dual Use Among Adult Employees in the US Workplace

**DOI:** 10.5888/pcd17.190217

**Published:** 2020-02-20

**Authors:** Christine M. Kava, Peggy A. Hannon, Jeffrey R. Harris

**Affiliations:** 1 Health Promotion Research Center, Department of Health Services, University of Washington, Seattle, Washington

## Abstract

**Introduction:**

Evidence-based interventions for tobacco control in the US workplace can reach a large audience. The purpose of our study was to explore the prevalence and determinants of type of tobacco use (ie, cigarettes only, e-cigarettes only, or dual use) among adult employees in the United States and to examine type of use by state.

**Methods:**

We used data from the 2017 Behavioral Risk Factor Surveillance System to examine the prevalence of cigarette use, e-cigarette use, dual use, and quit attempts. We used multinomial logistic regression to examine the relationships between sociodemographic characteristics and type of tobacco product used, and we estimated adjusted prevalence.

**Results:**

Approximately 17% of respondents were current smokers, 5% were current e-cigarette users, and 2% were dual users. E-cigarette-only and dual use were generally highest among young (aged 18–24), male, and less-educated respondents and lower for respondents who identified as black, Asian/Native Hawaiian/Pacific Islander, or Hispanic than for white respondents. Cigarette-only and dual use were higher for respondents who did not have health care coverage. Prevalence by state of e-cigarette use only ranged from 1.2% (Vermont) to 3.9% (Arkansas), whereas the prevalence of dual use ranged from 0.6% (District of Columbia) to 4.0% (Oklahoma).

**Conclusion:**

Prevalence of cigarette, e-cigarette, and dual use varied by sociodemographic characteristics and by state. These findings can support targeting of specific populations when designing and implementing evidence-based interventions for tobacco control in workplace settings.

**Summary**

**What is already known on this topic?**

US smoking rates have steadily declined over time, but e-cigarette use and dual use are becoming increasingly popular. Increased worksite evidence-based interventions are still needed for tobacco control.

**What is added by this report?**

Employment type, age, sex, race/ethnicity, education, and health care coverage were associated with e-cigarette use and dual cigarette and e-cigarette use. Recent quit attempts were higher among dual users. Tobacco-product use varied by state.

**What are the implications for public health practice?**

These findings suggest the importance of targeting efforts when designing and implementing worksite interventions for tobacco control and cessation in the workplace.

## Introduction

Smoking is the leading cause of preventable death in the United States (1). Smoking rates have declined over time, but the dual use of e-cigarettes and regular cigarettes has become increasingly popular. Current evidence on e-cigarettes shows that these products have adverse effects on the cardiovascular system and pulmonary function (2,3). Evidence-based interventions for tobacco control can reduce tobacco use (4,5). With approximately 60% of US adults currently employed (6), the workplace offers a large audience for these interventions. According to recent data (7), less than 20% of worksites have a policy banning all tobacco use or offer cessation programs, indicating a need for increased tobacco control interventions in the workplace.

Identifying characteristics associated with tobacco use among adult employees can help guide program implementation efforts. Previous studies found differences in employee tobacco use, including use of e-cigarettes, by sociodemographic characteristics such as sex and race (8,9). Tobacco use also varies by state (10,11). Our study explored the prevalence and determinants of type of tobacco use (cigarette-only, e-cigarette-only, and dual) among US adult employees to better understand their relationship with sociodemographic characteristics and employment type. We also examined state-level differences in use and report and compare data on recent quit attempts among cigarette-only users and among dual users. Although evidence on whether e-cigarettes help with smoking cessation is mixed (12,13), dual users may be primed to use worksite interventions to support their cessation. 

## Methods

### Design and sample

We used data from the 2017 Behavioral Risk Factor Surveillance System (BRFSS) (14). BRFSS is a random-digit–dial telephone survey that collects state-level data on a wide range of health behaviors and conditions and on use of health care services. The survey is conducted annually among people aged 18 or older . The response rate for the 2017 BRFSS was 45% (15). Additional information on BRFSS, including survey design and methodology, is available elsewhere (14). The total sample size for the 2017 BRFSS was 450,016. Our study included respondents in 50 US states and the District of Columbia who indicated that they were currently employed (N = 221,264).

### Measures

**Smoking status.** Respondents were asked the following questions: 1) “Have you smoked at least 100 cigarettes in your entire life?” and 2) “Do you now smoke cigarettes every day, some days, or not at all?” We coded respondents who had smoked at least 100 cigarettes and currently smoked every day or some days as current smokers, respondents who had smoked 100 cigarettes but did not currently smoke as former smokers, and respondents who had not smoked at least 100 cigarettes as never smokers.

**E-cigarette use**. Respondents were asked 1) “Have you ever used an e-cigarette or other electronic “vaping” product, even just one time, in your entire life?” and 2) “Do you now use e-cigarettes or other electronic vaping products every day, some days, or not at all?” We coded respondents who had used e-cigarettes in their lifetime and currently used e-cigarettes as current e-cigarette users, respondents who had used e-cigarettes but did not currently use them as former e-cigarette users, and respondents who had never used them as never e-cigarette users.

**Type of tobacco use**. We created a measure for the type of tobacco product used that included the following categories: no tobacco use (no current cigarette or e-cigarette use), cigarettes only (current cigarette use but not e-cigarette use), e-cigarettes only (current e-cigarette use but not cigarette use), and dual use (current cigarette and e-cigarette use).

**Quit attempts**. Current smokers were asked the following question: “During the past 12 months, have you stopped smoking for 1 day or longer because you were trying to quit smoking?” We coded respondents as making a recent quit attempt if they answered yes to this question.

**Sociodemographic characteristics**. We included the following sociodemographic variables: age (18–24, 25–44, 45–64, ≥65), annual household income (<$15,000, $15,000–$24,999, $25,000–$34,999, $35,000–$49,999, ≥$50,000); education (less than high school, high school graduate, some college, college graduate), employment type (employed for wages or self-employed), sex (male or female), race/ethnicity (white, black, American Indian/Alaska Native, Asian/Native Hawaiian/Pacific Islander, other [other race or multiracial], or Hispanic), and whether the respondent had any kind of health care coverage (yes or no).

### Data analysis

We conducted data analysis in Stata version 15 (Stata Corp LLC). To account for the complex survey design, we used the weight, strata, and cluster variables included in the BRFSS data set. We centered strata with only 1 primary sampling unit at the grand mean. We calculated descriptive statistics and produced 95% confidence interval estimates for general sociodemographic characteristics and tobacco-use behavior; we examined quit-attempt behavior separately for cigarette-only users and dual users.

We conducted a multinomial logistic regression to examine the adjusted relationships among sociodemographic characteristics and the type of tobacco product used. Given a moderate and significant correlation between education and income (ρ = 0.39; *P* < .001), we excluded income from multivariable analysis, which had a larger percentage of missing data. After running the multinomial logistic regression, we calculated predictive margins (16) to estimate the adjusted prevalence of types of tobacco use. To examine differences by state we calculated state-level prevalence, with confidence intervals, of the types of tobacco use.

## Results

Approximately 17% of respondents were current smokers, and 22% were former smokers (Table 1). Five percent were current e-cigarette users, 2% were dual users, and 18% were former e-cigarette users. Approximately 14% currently used cigarettes only, and 3% used e-cigarettes only. Approximately one-quarter (23%) of respondents had ever tried e-cigarettes. Recent quit attempts were higher among dual users (70%) than cigarette-only users (56%), and nearly two-thirds (61%) of e-cigarette-only users were former smokers. Most respondents were male (55%), white (63%), had at least some college education (64%), and had an annual household income of $50,000 or greater (60%). Eighty-four percent were employed for wages and 16% were self-employed; most (87%) had health care coverage.

**Table 1:** Tobacco Use Among Adult Employees in the US Workplace (N = 221,264), Behavioral Risk Factor Surveillance System, 2017^a^

Variable	n	% (95% CI)
**Smoking status**		
Current	31,356	16.5 (16.2–16.9)
Former	50,572	21.6 (21.3–22.0)
Never	130,016	61.9 (61.5–62.3)
**E-cigarette use**		
Current user	7,600	4.8 (4.6–5.0)
Former user	30,697	17.8 (17.5–18.2)
Never user	172,495	77.4 (77.1–77.8)
**Type of tobacco use**		
None	175,063	81.1 (80.8–81.5)
Cigarettes only	27,216	14.1 (13.8–14.4)
E-cigarettes only	3,850	2.5 (2.3–2.6)
Dual use	3,716	2.3 (2.2–2.4)
**Recent quit attempt among cigarette-only users^b^**		
No	12,463	43.7 (42.5–44.9)
Yes	14,655	56.3 (55.1–57.5)
**Recent quit attempt among dual users^b^**		
No	1,194	30.4 (27.6–33.3)
Yes	2,509	69.6 (66.7–72.4)
**% E-cigarette-only users who identified as former smokers**	2,600	60.6 (57.7–63.5)

Abbreviation: CI, confidence interval.

a Percentages are weighted.

b Asked only among current smokers. Recent quit attempts were those within the past 12 months.

Overall, the odds of cigarette-only, e-cigarette-only, and dual use compared with no tobacco use decreased as age and education increased (Table 2). Odds of cigarette-only use were higher among adults aged 25 to 44 and 45 to 64 than among those aged 18 to 24. Women were less likely than men to use tobacco in any form, as were respondents who self-identified as black, Asian/Native Hawaiian/Pacific Islander, or Hispanic compared with those who self-identified as white. In contrast, those classified as other race and American Indian/Alaska Native were more likely to be cigarette-only users; other-race respondents also had a greater likelihood of e-cigarette-only use. Respondents without health care coverage had higher odds of cigarette-only and dual use. Respondents who identified as self-employed had lower odds of being an e-cigarette-only user than those employed for wages.

**Table 2:** Odds Ratios and Adjusted Prevalence of Type of Tobacco Use Among Adult Employees in the US Workplace (N = 221,264), Behavioral Risk Factor Surveillance System, 2017^a^

Variable	Type of Tobacco Use^b^					
	Cigarettes Only		E-Cigarettes Only		Dual Use	
	OR	Adjusted % (95% CI)	OR	Adjusted % (95% CI)	OR	Adjusted % (95% CI)
**Employment type**						
Employed for wages	1 [Reference]	14.3 (14.0–14.6)	1 [Reference]	2.5 (2.3–2.6)	1 [Reference]	2.2 (2.2–2.4)
Self-employed	0.9	13.4 (12.6–14.3)	0.8^c^	2.1 (1.8–2.4)	1.2	2.8 (2.3–3.3)
**Age, y**						
18–24	1 [Reference]	10.6 (9.7–11.5)	1 [Reference]	6.8 (6.1–7.6)	1 [Reference]	3.6 (3.1–4.2)
25–44	1.7^c^	17.0 (16.4–17.5)	0.4^c^	2.7 (2.5–3.0)	0.9	3.0 (2.7–3.2)
45–64	1.1^c^	13.1 (12.6–13.5)	0.1^c^	1.1 (0.9–1.2)	0.4^c^	1.4 (1.2–1.5)
≥65	0.6^c^	7.7 (6.9–8.4)	0.0^c^	0.3 (0.2–0.4)	0.2^c^	0.7 (0.4–1.0)
**Sex**						
Male	1 [Reference]	14.9 (14.5–15.3)	1 [Reference]	3.0 (2.8–3.3)	1 [Reference]	2.5 (2.3–2.7)
Female	0.8^c^	13.1 (12.7–13.6)	0.5^c^	1.6 (1.5–1.8)	0.8^c^	2.1 (1.9–2.3)
**Race/ethnicity**						
White	1 [Reference]	16.5 (16.1–16.9)	1 [Reference]	2.9 (2.8–3.1)	1 [Reference]	2.9 (2.7–3.1)
Black	0.8^c^	13.8 (12.9–14.7)	0.5^c^	1.6 (1.2–2.0)	0.5^c^	1.7 (1.3–2.2)
American Indian/Alaska Native	1.4^c^	21.1 (18.5–23.8)	1.2	3.3 (2.0–4.7)	1.1	3.0 (1.9–4.0)
Asian/Native Hawaiian/Pacific Islander	0.6^c^	11.7 (9.7–13.7)	0.5^c^	1.7 (1.1–2.3)	0.5^c^	1.6 (1.0–2.2)
Other^d^	1.2^c^	18.1 (16.2–20.0)	1.4^c^	3.8 (2.8–4.7)	1.2	3.3 (2.3–4.3)
Hispanic	0.4^c^	8.2 (7.5–9.0)	0.4^c^	1.4 (1.1–1.6)	0.3^c^	1.2 (0.9–1.5)
**Education**						
Less than high school	1 [Reference]	28.4 (26.5–30.2)	1 [Reference]	2.1 (1.5–2.8)	1 [Reference]	3.2 (2.5–4.0)
High school graduate	0.6^c^	20.3 (19.6–21.0)	1.3	3.0 (2.7–3.3)	0.9	3.2 (2.9–3.5)
Some college	0.4^c^	14.8 (14.2–15.3)	1.1	2.9 (2.6–3.2)	0.7^c^	2.8 (2.5–3.1)
College graduate	0.1^c^	5.4 (5.1–5.7)	0.5^c^	1.5 (1.3–1.6)	0.2^c^	0.9 (0.8–1.0)
**Health care coverage**						
Yes	1 [Reference]	13.1 (12.8–13.5)	1 [Reference]	2.5 (2.3–2.6)	1 [Reference]	2.2 (2.0–2.3)
No	1.7^c^	19.9 (18.8–20.9)	1.0	2.3 (1.9–2.6)	1.7^c^	3.2 (2.7–3.6)

Abbreviations: CI, confidence interval; OR, odds ratio.

a Percentages are weighted. Odds ratios were produced by using multinomial logistic regression. We used predictive margins to calculate the adjusted prevalence.

b Reference is no tobacco use.

c Significant at *P* < .05.

d Other race or multiracial.

We calculated the adjusted prevalence for the type of tobacco product used (Table 2) and the unadjusted prevalence (Appendix). The prevalence of cigarette-only use varied the most by education: 28% of respondents with less than high school education used cigarettes only, whereas only 5% of college graduates were cigarette-only users. E-cigarette-only use was highest for respondents aged 18 to 24 (7%), followed by other-race respondents (4%). Age showed the largest percentage difference in dual use: 4% of respondents aged 18 to 24 were dual users compared with less than 1% for respondents aged 65 or older.

**State differences**. The prevalence of cigarette-only use ranged from 7.2% in Utah to 21.5% in West Virginia (Table 3). The states with the lowest prevalence of e-cigarette-only use were Vermont (1.2%), South Dakota (1.4%), and District of Columbia (1.6%). The states with the highest prevalence of e-cigarette-only use were Arkansas (3.9%), Oklahoma (3.7%), and Utah (3.7%). Dual use was lowest in District of Columbia (0.6%), Alaska (1.4%), and California (1.5%), and highest in Oklahoma (4.0%), West Virginia (3.6%), and Indiana (3.3%).

**Table 3:** Type of Tobacco Use, by Type of Tobacco Product and by State, Among Adult Employees in the US Workplace, Behavioral Risk Factor Surveillance System, 2017^a^

State	Cigarettes Only (N = 27,216)	E-cigarettes Only (N = 3,850)	Dual Use (N = 3,716)
Alabama	17.6 (15.7–19.6)	2.6 (1.8–3.7)	2.8 (2.1–3.9)
Alaska	17.2 (14.4–20.5)	2.1 (1.1–4.1)	1.4 (0.7–2.9)
Arizona	12.9 (11.9–14.0)	3.2 (2.7–3.8)	2.4 (2.0–3.0)
Arkansas	19.6 (16.5–23.2)	3.9 (2.4–6.2)	3.0 (1.8–5.1)
California	10.8 (9.5–12.1)	2.1 (1.7–2.7)	1.5 (1.1–2.0)
Colorado	12.4 (11.3–13.7)	3.2 (2.6–3.9)	2.7 (2.2–3.4)
Connecticut	11.3 (10.0–12.7)	1.6 (1.2–2.2)	1.7 (1.3–2.3)
Delaware	14.4 (12.3–16.8)	2.4 (1.5–3.8)	2.8 (1.9–3.9)
District of Columbia	10.6 (8.9–12.5)	1.6 (1.0–2.6)	0.6 (0.3–1.0)
Florida	13.9 (12.4–15.7)	2.3 (1.7–3.0)	2.2 (1.7–2.9)
Georgia	14.8 (13.2–16.7)	2.2 (1.7–3.1)	2.1 (1.5–3.0)
Hawaii	11.8 (10.5–13.2)	3.4 (2.7–4.3)	1.8 (1.3–2.4)
Idaho	12.7 (10.9–14.7)	3.0 (2.1–4.3)	2.7 (1.9–3.8)
Illinois	13.8 (12.2–15.6)	2.4 (1.8–3.4)	2.5 (1.7–3.6)
Indiana	18.9 (17.6–20.2)	3.2 (2.6–3.9)	3.3 (2.7–4.1)
Iowa	15.9 (14.6–17.2)	1.9 (1.5–2.6)	1.7 (1.3–2.2)
Kansas	15.2 (14.4–16.1)	2.7 (2.3–3.1)	2.4 (2.0–2.8)
Kentucky	20.6 (18.6–22.7)	3.0 (2.2–4.2)	3.1 (2.3–4.1)
Louisiana	19.9 (17.7–22.2)	2.3 (1.7–3.2)	2.1 (1.4–3.0)
Maine	14.9 (13.3–16.6)	1.9 (1.4–2.8)	2.3 (1.6–3.1)
Maryland	11.7 (10.4–13.0)	1.9 (1.4–2.5)	1.6 (1.1–2.1)
Massachusetts	10.9 (9.3–12.7)	1.6 (1.0–2.6)	1.5 (1.0–2.3)
Michigan	17.2 (15.9–18.7)	2.9 (2.3–3.7)	2.4 (1.9–3.0)
Minnesota	13.3 (12.4–14.2)	1.8 (1.5–2.2)	1.8 (1.4–2.2)
Mississippi	18.8 (16.4–21.6)	2.1 (1.3–3.4)	2.8 (1.9–4.0)
Missouri	17.5 (15.8–19.3)	2.8 (2.1–3.8)	2.7 (2.0–3.6)
Montana	16.9 (15.1–18.9)	2.1 (1.4–3.1)	1.7 (1.1–2.5)
Nebraska	13.5 (12.4–14.7)	1.9 (1.4–2.5)	1.8 (1.4–2.3)
Nevada	16.4 (13.9–19.3)	3.5 (2.3–5.2)	2.3 (1.3–3.9)
New Hampshire	13.0 (11.1–15.1)	2.4 (1.5–3.7)	2.3 (1.5–3.6)
New Jersey	11.5 (10.1–13.0)	2.2 (1.7–3.0)	2.6 (1.9–3.6)
New Mexico	14.2 (12.4–16.2)	3.3 (2.3–4.7)	2.3 (1.6–3.2)
New York	12.4 (11.2–13.6)	2.2 (1.7–2.8)	1.7 (1.3–2.3)
North Carolina	13.8 (12.1–15.7)	2.5 (1.8–3.5)	2.8 (2.0–4.0)
North Dakota	16.9 (15.3–18.5)	2.3 (1.6–3.2)	2.3 (1.6–3.1)
Ohio	17.7 (16.2–19.2)	2.8 (2.2–3.6)	2.7 (2.2–3.5)
Oklahoma	16.3 (14.6–18.2)	3.7 (2.9–4.7)	4.0 (3.1–5.2)
Oregon	13.6 (12.1–15.3)	2.8 (2.1–3.7)	2.7 (2.0–3.7)
Pennsylvania	16.0 (14.4–17.8)	2.3 (1.7–3.1)	2.9 (2.3–3.8)
Rhode Island	13.5 (11.6–15.8)	2.5 (1.8–3.6)	1.6 (1.0–2.4)
South Carolina	17.4 (16.0–19.0)	3.0 (2.3–3.9)	1.8 (1.4–2.4)
South Dakota	18.6 (16.3–21.1)	1.4 (0.8–2.6)	3.2 (2.0–5.2)
Tennessee	19.0 (17.0–21.3)	2.8 (2.0–3.9)	2.8 (2.0–3.9)
Texas	13.4 (11.7–15.3)	2.4 (1.6–3.4)	3.0 (2.2–4.1)
Utah	7.2 (6.4–8.1)	3.7 (3.0–4.4)	2.0 (1.5–2.5)
Vermont	14.3 (12.7–16.1)	1.2 (0.7–2.0)	1.7 (1.0–2.8)
Virginia	13.9 (12.6–15.3)	2.7 (2.1–3.5)	2.4 (1.8–3.1)
Washington	11.9 (10.9–13.0)	2.4 (1.9–2.9)	1.7 (1.3–2.2)
West Virginia	21.5 (19.5–23.8)	2.4 (1.7–3.5)	3.6 (2.7–4.8)
Wisconsin	14.2 (12.5–15.9)	2.8 (2.0–3.9)	2.0 (1.4–2.8)
Wyoming	16.4 (14.6–18.5)	3.0 (2.1–4.2)	2.9 (2.1–4.1)

Abbreviation: CI, confidence interval.

a Values are percentage (95% confidence interval). Percentages are weighted. Data are for current tobacco users.

## Discussion

Tobacco use, especially e-cigarette and dual use, tended to be most prevalent among young white males with less than a high school education. These findings are consistent with previous studies (9) and suggest the importance of targeting these populations when designing worksite programs for tobacco cessation. One approach could be to target policy, communication, and cessation program efforts within occupations and industries where a larger proportion of these populations reside. For example, according to data from the 2018 Current Population Survey, people employed in construction are 90% male and 88% white (17).

Previous studies have suggested that worksite culture, social norms (eg, coworker discouragement of quitting), and job stress may contribute to higher rates of tobacco use within construction and similar industries and occupations (18–20). Given this, efforts to reduce tobacco use should address both individual and organizational factors that contribute to a higher prevalence. Examples of evidence-based interventions for tobacco control include quitline counseling, nicotine-replacement therapy for cessation, and strong tobacco-free worksite policies (4,5). Incorporating strategies to reduce work stress (eg, increasing job control) may also help with cessation efforts (21).

Respondents with health care coverage had a lower prevalence of cigarette-only and dual use than those without. The Guide to Community Preventive Services recommends reducing out-of-pocket costs for evidence-based cessation treatments to reduce tobacco use (5). Employers can offer treatment benefits via employer-sponsored health insurance to help reduce treatment costs or copayments (5,22). Since the Affordable Care Act mandated the provision of preventive services, offering these benefits is now a requirement for employers with 50 or more full-time employees. These provisions have also helped to increase insurance coverage among the self-employed (23), a group not traditionally reached by worksite interventions. Smaller worksites are less likely to offer health insurance (24) and could be prioritized for intervention efforts to ensure that employees have access to tobacco control interventions. Group purchasing via trade associations and unions to increase insurance and cessation-program access is one possibility (24). States and worksites that have not expanded Medicaid or have low insurance coverage could also be urged to invest in and publicize state quitlines.

The prevalence of current cigarette (17%) and e-cigarette use (5%) found here were similar to a previous study among working adults reporting 15% and 4%, respectively (9). Consistent with previous studies (25), recent quit attempts were higher among dual users (70%) than cigarette-only users (56%). We also found that more than half of all e-cigarette-only users identified as former smokers. Taken together, these findings suggest that dual users may be using e-cigarettes as a smoking cessation aid and align with previous studies that found a higher readiness to quit among dual users (26). These results provide evidence in favor of targeting dual users when implementing cessation interventions, such as improved access to quitlines, in the worksite.

The prevalence of cigarette-only, e-cigarette-only, and dual use varied by state, and states with the lowest cigarette use did not always have the lowest e-cigarette or dual use. For example, although Utah had the lowest prevalence of cigarette-only users, prevalence of e-cigarette use was among the highest in the country. Additional research is needed to understand these relationships, but our findings provide insight into which states could benefit the most from comprehensive worksite policies and programs, for example, by prohibiting tobacco use both indoors and outdoors on campuses or explicitly addressing e-cigarette use in policy language.

Comprehensive clean indoor air laws at the state level can provide environmental support for cessation among employees. Because these laws restrict tobacco use in enclosed spaces, they have direct implications for worksite policy. Although nearly all states have some city or county ordinances that ban smoking in nonhospitality worksites, bars, and restaurants, only 27 states have enacted these ordinances at the state level (27). Excise tax rates on cigarettes, which vary widely by state (from $0.17 to $4.35 per pack) (28), can also affect smoking behavior. We found that employee smoking was highest in West Virginia, a state with a relatively low excise tax on cigarettes ($1.20/pack) (28) and fewer provisions on indoor smoking at the state level (27,29). Although Utah, the state with the highest e-cigarette prevalence among employees, is one of 17 states to restrict e-cigarette use in nonhospitality worksites, bars, and restaurants at the state level (30), it is not one of the 21 states that have enacted an excise tax on e-cigarettes (31). These data suggests that opportunities exist to improve these policies in an effort to reduce tobacco use.

This study had limitations. Data on occupation and industry were not publicly available in the BRFSS data set, which limited our ability to make worksite policy and program recommendations based on these data. The 2017 BRFSS had limited data on e-cigarette use; thus, it was not possible to assess whether respondents were experimenters or more frequent and long-term e-cigarette users. Future studies should collect more detailed data on length, intensity, and frequency of tobacco use. Study strengths were its large sample size (N = 221,264), strong sampling design, and a detailed examination of the determinants of tobacco use by sociodemographic characteristics and by state.

Findings from our study expand understanding of tobacco-product use among employees and have direct implications for worksite implementation of interventions for tobacco control. Practitioners and researchers can apply these findings to design and implement interventions and to select worksite populations likely to have employees who will benefit. The findings from our study can also inform which employee groups to prioritize when designing worksite interventions, and which states could benefit most from strong clean indoor air laws to protect worksites and their employees from the negative consequences of tobacco use.

## Appendix

**Supplemental Table:** Unadjusted Prevalence for Type of Tobacco Use Among Employed Adults in the US Workplace (N = 221,264), Behavioral Risk Factor Surveillance System, 2017^a^

Variable	Tobacco Type		
	Cigarettes Only	E-Cigarettes Only	Dual Use
**Employment type**			
Employed for wages	14.0 (13.7–14.4)	2.6 (2.4–2.8)	2.2 (2.1–2.4)
Self-employed	14.6 (13.7–15.5)	1.8 (1.5–2.1)	2.6 (2.2–3.1)
**Age, y**			
18–24	12.4 (11.4–13.4)	7.6 (6.8–8.4)	4.1 (3.5–4.7)
25–44	16.4 (15.9–17.0)	2.6 (2.4–2.9)	2.8 (2.6–3.1)
45–64	13.1 (12.7–13.5)	1.1 (1.0–1.2)	1.4 (1.3–1.6)
≥65	7.2 (6.6–7.9)	0.4 (0.2–0.6)	0.7 (0.5–1.0)
**Sex**			
Male	15.8 (15.3–16.3)	3.2 (3.0–3.4)	2.6 (2.4–2.8)
Female	12.0 (11.6–12.5)	1.6 (1.4–1.7)	1.9 (1.7–2.1)
**Race/ethnicity**			
White	14.7 (14.3–15.0)	2.8 (2.6–3.0)	2.6 (2.4–2.8)
Black	14.5 (13.6–15.5)	1.7 (1.3–2.2)	1.9 (1.5–2.5)
American Indian/Alaska Native	23.4 (20.5–26.5)	3.9 (2.6–5.9)	3.3 (2.4–4.7)
Asian/Native Hawaiian/Pacific Islander	8.1 (6.7–9.7)	1.5 (1.0–2.1)	1.2 (0.8–1.7)
Other	17.7 (15.8–19.7)	4.3 (3.3–5.5)	3.5 (2.6–4.7)
Hispanic	13.1 (12.2–14.2)	1.8 (1.4–2.1)	1.8 (1.4–2.2)
**Education**			
Less than high school	26.2 (24.6–27.9)	1.9 (1.5–2.5)	3.0 (2.5–3.6)
High school graduate	20.3 (19.6–21.0)	3.6 (3.2–3.9)	3.4 (3.1–3.7)
Some college	14.8 (14.3–15.4)	3.0 (2.7–3.3)	2.8 (2.5–3.1)
College graduate	5.4 (5.2–5.7)	1.3 (1.2–1.4)	0.9 (0.8–1.0)
**Annual household income, $**			
Less than 15,000	21.3 (19.4–23.2)	2.0 (1.5–2.7)	2.7 (2.1–3.5)
15,000–24,999	22.8 (21.7–24.0)	2.6 (2.2–3.1)	3.9 (3.4–4.5)
25,000–34,999	20.1 (18.8–21.4)	3.0 (2.5–3.5)	2.9 (2.4–3.4)
35,000–49,999	18.6 (17.5–19.7)	2.6 (2.2–3.0)	3.1 (2.6–3.5)
≥50,000	10.0 (9.7–10.4)	2.3 (2.1–2.4)	1.7 (1.5–1.9)
**Health care coverage**			
No	24.8 (23.6–26.0)	2.6 (2.3–3.1)	3.9 (3.4–4.5)
Yes	12.6 (12.3–12.9)	2.4 (2.3–2.6)	2.1 (1.9–2.2)

Abbreviations: CI, confidence interval.

^a ^Percentages are weighted. Values are percentage (95% confidence interval).

## References

[1] US Department of Health and Human Services. The health consequences of smoking — 50 years of progress: a report of the Surgeon General. Atlanta (GA): US Department of Health and Human Services, Centers for Disease Control and Prevention, National Center for Chronic Disease Prevention and Health Promotion, Office on Smoking and Health; 2014.

[2] Glantz SA , Bareham DW . E-cigarettes: use, effects on smoking, risks, and policy implications. Annu Rev Public Health 2018;39:215–35. 10.1146/annurev-publhealth-040617-013757 29323609 PMC6251310

[3] Bhatta DN , Glantz SA . Electronic cigarette use and myocardial infarction among adults in the US Population Assessment of Tobacco and Health. J Am Heart Assoc 2019;8(12):e012317. 10.1161/JAHA.119.012317 31165662 PMC6645634

[4] Cahill K , Lancaster T . Workplace interventions for smoking cessation. Cochrane Database Syst Rev 2014;(2):CD003440. 24570145 10.1002/14651858.CD003440.pub4 PMC11285308

[5] Guide to Community Preventive Services. Reducing tobacco use and secondhand smoke exposure. https://www.thecommunityguide.org/topic/tobacco. Accessed April 25, 2019.

[6] Bureau of Labor Statistics. Labor force statistics from the Current Population Survey: employment status of the civilian noninstitutional population, 1947 to date. https://www.bls.gov/cps/cpsaat01.pdf. Accessed April 25, 2019.

[7] Linnan LA , Cluff L , Lang JE , Penne M , Leff MS . Results of the Workplace Health in America Survey. Am J Health Promot 2019;33(5):652–65. 10.1177/0890117119842047 31007038 PMC6643274

[8] Syamlal G , Jamal A , King BA , Mazurek JM . Electronic cigarette use among working adults — United States, 2014. MMWR Morb Mortal Wkly Rep 2016;65(22):557–61. 10.15585/mmwr.mm6522a1 27281058

[9] Syamlal G , King BA , Mazurek JM . Tobacco use among working adults — United States, 2014–2016. MMWR Morb Mortal Wkly Rep 2017;66(42):1130–5. 10.15585/mmwr.mm6642a2 29072865 PMC5689107

[10] Mirbolouk M , Charkhchi P , Kianoush S , Uddin SMI , Orimoloye OA , Jaber R , Prevalence and distribution of e-cigarette use among US adults: Behavioral Risk Factor Surveillance System, 2016. Ann Intern Med 2018;169(7):429–38. 10.7326/M17-3440 30167658 PMC10534294

[11] Hu SS , Homa DM , Wang T , Gomez Y , Walton K , Lu H , State-specific patterns of cigarette smoking, smokeless tobacco use, and e-cigarette use among adults — United States, 2016. Prev Chronic Dis 2019;16:E17. 10.5888/pcd16.180362 30730828 PMC6395075

[12] Kalkhoran S , Glantz SA . E-cigarettes and smoking cessation in real-world and clinical settings: a systematic review and meta-analysis. Lancet Respir Med 2016;4(2):116–28. 10.1016/S2213-2600(15)00521-4 26776875 PMC4752870

[13] Rahman MA , Hann N , Wilson A , Mnatzaganian G , Worrall-Carter L . E-cigarettes and smoking cessation: evidence from a systematic review and meta-analysis. PLoS One 2015;10(3):e0122544. 10.1371/journal.pone.0122544 25822251 PMC4378973

[14] Centers for Disease Control and Prevention. Behavioral Risk Factor Surveillance System. 2019; https://www.cdc.gov/brfss/. Accessed January 24, 2020.

[15] Centers for Disease Control and Prevention. The Behavioral Risk Factor Surveillance System 2017 summary data quality report. https://www.cdc.gov/brfss/annual_data/2017/pdf/2017-sdqr-508.pdf. Accessed April 26, 2019.

[16] Graubard BI , Korn EL . Predictive margins with survey data. Biometrics 1999;55(2):652–9. 10.1111/j.0006-341X.1999.00652.x 11318229

[17] Bureau of Labor Statistics. Employed persons by detailed industry, sex, race, and Hispanic or Latino ethnicity. https://www.bls.gov/cps/cpsaat18.htm. Accessed April 30, 2019.

[18] Sorensen G , Barbeau E , Hunt MK , Emmons K . Reducing social disparities in tobacco use: a social-contextual model for reducing tobacco use among blue-collar workers. Am J Public Health 2004;94(2):230–9. 10.2105/AJPH.94.2.230 14759932 PMC1448233

[19] Sorensen G , Quintiliani L , Pereira L , Yang M , Stoddard A . Work experiences and tobacco use: findings from the gear up for health study. J Occup Environ Med 2009;51(1):87–94. 10.1097/JOM.0b013e31818f69f8 19136877

[20] Syamlal G , King BA , Mazurek JM . Tobacco product use among workers in the construction industry, United States, 2014–2016. Am J Ind Med 2018;61(11):939–51. 10.1002/ajim.22907 30229974 PMC6350769

[21] Kouvonen A , Kivimäki M , Virtanen M , Pentti J , Vahtera J . Work stress, smoking status, and smoking intensity: an observational study of 46,190 employees. J Epidemiol Community Health 2005;59(1):63–9. 10.1136/jech.2004.019752 15598729 PMC1763376

[22] American Heart Association. Tobacco control in the workplace. http://playbook.heart.org/wp-content/uploads/2015/09/Tobacco-Policy-Summary-FINAL.pdf. Accessed January 24, 2020.

[23] Decker SL , Moriya AS , Soni A . Coverage for self-employed and others without employer offers increased after 2014. Health Aff (Millwood) 2018;37(8):1238–42. 10.1377/hlthaff.2017.1663 30080453

[24] Harris JR , Huang Y , Hannon PA , Williams B . Low-socioeconomic status workers: their health risks and how to reach them. J Occup Environ Med 2011;53(2):132–8. 10.1097/JOM.0b013e3182045f2c 21270663

[25] Peltier MR , Waters AF , Roys MR , Stewart SA , Waldo KM , Copeland AL . Dual users of e-cigarettes and cigarettes have greater positive smoking expectancies than regular smokers: a study of smoking expectancies among college students. J Am Coll Health 2019;1–6. 10.1080/07448481.2019.1590373 30908173 PMC11013953

[26] Fahey MC , Bursac Z , Ebbert JO , Klesges RC , Little MA . Prevalence and correlates of dual tobacco use in cancer survivors. Cancer Causes Control 2019;30(3):217–23. 10.1007/s10552-019-1132-6 30671688

[27] American Nonsmokers’ Rights Foundation. US 100% smokefree laws in non-hospitality workplaces and restaurants and bars. https://no-smoke.org/wp-content/uploads/pdf/WRBLawsMap.pdf. Accessed January 31, 2020.

[28] Centers for Disease Control and Prevention. Map of excise tax rates on cigarettes. https://www.cdc.gov/statesystem/excisetax.html. Accessed June 18, 2019.

[29] Centers for Disease Control and Prevention. Map of smokefree indoor air — private worksites, restaurants, and bars. https://www.cdc.gov/statesystem/smokefreeindoorair.html. Accessed June 18, 2019.

[30] American Nonsmokers’ Rights Foundation. States and municipalities with laws regulating use of electronic cigarettes. http://no-smoke.org/wp-content/uploads/pdf/ecigslaws.pdf. Accessed January 31, 2020.

[31] Campaign for Tobacco-Free Kids. State excise tax rates for non-cigarette tobacco products. https://www.tobaccofreekids.org/assets/factsheets/0169.pdf. Accessed January 31, 2020.

